# A giant cause of a low pressure headache

**DOI:** 10.1186/1129-2377-14-S1-P165

**Published:** 2013-02-21

**Authors:** S Miller, J Overell, R Jampana, G Gorrie, A Tyagi, C Gilles, M McKenzie

**Affiliations:** 1 Glasgow Headache Group, Institute of Neurological Sciences, Southern General Hospital, Glasgow, UK; 2 Department of Neurology, Institute of Neurological Sciences, Southern General Hospital, Glasgow, UK; 3 Department of Neuroradiology, Institute of Neurological Sciences, Southern General Hospital, Glasgow, UK

## 

We present an unusual case of low pressure headaches in a patient with Marfan’s Syndrome caused by a giant anterior sacral meningocele. The patient, a lady with Marfan’s Syndrome and previous cardiac and ocular complications, presented initially with recurrent postural headaches, worse on standing, occurring over a 5 year period. During these periods she would develop pulsatile tinnitus on standing and also felt the headaches improved when she breathed in. CT brain scans, MRI scans of head and neck and recurrent lumbar punctures done during this period where normal although opening pressures were not recorded. Since 2007 she has had a chronic daily headache with no associated features. Neurological examinations were normal throughout her presentations. A MRI of her lumboscaral spine was performed in 2011 with showed a massive fluid filled structure in the pelvis and a diagnosis of a giant dural ectasia or anterior sacral meningocele was made. Anterior sacral meningoceles are an uncommon congenital abnormality consisting of a spinal fluid filled sac in the pelvis communicating with the subarachnoid space. They are associated with Currarino’s syndrome (presacral mass, a sacral defect and an anorectal malformation) and Marfan’s syndrome. Patients most often present with abdominal symptoms, symptoms of cauda equina or with headaches (both high and low pressure). Neurological symptoms may respond to surgical treatment of the meningocele.

